# Kinetics of LYVE-1-positive M2-like macrophages in developing and repairing dental pulp in vivo and their pro-angiogenic activity in vitro

**DOI:** 10.1038/s41598-022-08987-3

**Published:** 2022-03-25

**Authors:** Thoai Quoc Kieu, Kento Tazawa, Nobuyuki Kawashima, Sonoko Noda, Mayuko Fujii, Keisuke Nara, Kentaro Hashimoto, Peifeng Han, Takashi Okiji

**Affiliations:** 1 Graduate School of Medical and Dental Sciences, Tokyo Medical and Dental University (TMDU), 1-5-45 Yushima, Bunkyo-ku, Tokyo, 113-8549 Japan; 2 Department of Cariology, Restorative Sciences, and Endodontics, University of Michigan School of Dentistry, 1011 N University Avenue, Ann Arbor, MI 48109 USA

**Keywords:** Pulpitis, Dental pulp

## Abstract

Tissue-resident macrophages expressing lymphatic vessel endothelial hyaluronan receptor-1 (LYVE-1) are found in multiple tissues and organs. We aimed to evaluate the dynamics and biological functions of LYVE-1^+^ macrophages in dental pulp during post-injury tissue remodeling. Immunofluorescence staining of mouse embryos revealed that LYVE-1^+^ macrophages colonized dental pulp before birth. In mature rat molar dental pulp, LYVE-1^+^ macrophages were the main subset of macrophages expressing CD163, an M2 marker, and were distributed throughout the tissue. In response to dental pulp injury induced by cavity preparation, LYVE-1^+^ macrophages quickly disappeared from the affected area of the pulp and gradually repopulated during the wound healing process. RAW264.7 mouse macrophages cultured with a mixture of macrophage colony-stimulating factor, interleukin-4, and dexamethasone increased LYVE-1 expression, whereas lipopolysaccharide-stimulation decreased LYVE-1 expression. Enforced expression of *Lyve1* in RAW264.7 cells resulted in increased mRNA expression of matrix metalloproteinase 2 (*Mmp2*), *Mmp9*, and vascular endothelial growth factor A (*Vegfa*). *Lyve1*-expressing macrophages promoted the migration and tube formation of human umbilical vein endothelial cells. In conclusion, LYVE-1^+^ tissue-resident M2-like macrophages in dental pulp showed dynamism in response to pulp injury, and possibly play an important role in angiogenesis during wound healing and tissue remodeling.

## Introduction

Tissue-resident macrophages populate every tissue, playing important roles in support of tissue development, homeostasis, and remodeling, which makes them attractive therapeutic targets^1–4^. The functions and phenotypes of tissue-resident macrophages are extremely heterogeneous, depending on the ontogeny, local microenvironment, and inflammation status of the tissue in which they reside^5^. It is now understood that most tissue-resident macrophage populations are established during embryonic development from yolk sac or fetal liver precursors and maintain themselves throughout adulthood by self-renewal with minimal contribution from blood-circulating monocytes^6–8^. According to the influence of tissue-specific cues, precursors recruited to the tissue differentiate into specialized tissue-resident macrophages that are integral to the function and homeostasis of the tissue^9^. In response to external stimuli, the constantly fluctuating local tissue microenvironment results in a certain degree of dynamism of tissue-resident macrophages^5,10^. As a consequence of ontogenic and tissue niche heterogeneity, each tissue contains multiple phenotypically distinct populations of tissue-resident macrophages^2^. However, the precise components of tissue-resident macrophages in different tissue niches remain to be identified. Most tissue-resident macrophages have been classified as alternatively activated (M2) macrophages that perform fundamental roles in the resolution of inflammation and tissue repair after infections or injuries^2,11^.

Lymphatic vessel endothelial hyaluronan receptor-1 (LYVE-1) is a homolog of CD44 and was originally identified as a lymphatic vessel-specific marker^12^. Recent works have reported the existence of tissue-resident macrophages expressing LYVE-1 in various tissues including heart, lung, dermis, fat, aorta, eyes, and meninges^13–16^. They co-express typical M2 macrophage markers (CD163, CD206) and play a wide range of homeostasis and tissue repair functions^13,14^. For example, in the murine lung, LYVE-1^+^ macrophages have been identified as the main population of interstitial macrophages that support blood vessel integrity under steady-state conditions^13^. In adipose tissue, recruitment of LYVE-1^+^ macrophages is essential for the formation of dense vascular networks, suggesting their ability to support angiogenesis^17^. Notably, LYVE-1^+^ macrophages also exhibit their dynamism in response to changes in their tissue niches. Resident arterial LYVE-1^+^ macrophages disappear from inflamed tissues and rapidly return to normal levels when the inflammation subsides^18^.

The dental pulp is susceptible to damage resulting from dental caries, dental trauma, and surgical procedures, amongst others. However, the dental pulp is well-equipped with several tissue-resident macrophage populations that can mount a defense response, mitigate inflammation, and accelerate the healing process^19–22^. Our previous study demonstrated the existence of a LYVE-1^+^ macrophage population in rat dental pulp tissue under steady-state conditions^23^. However, the biological characteristics of LYVE-1^+^ macrophages in the dental pulp remain to be determined. It is unclear when these cells start to colonize this tissue, and how they respond to exogenous stimuli and participate in the repair processes. Thus, the aims of the present study were to examine the existence and phenotype of LYVE-1^+^ macrophages in developing and mature dental pulp, to observe the kinetics of LYVE-1 macrophages after pulp injury, and to evaluate the biological roles of LYVE-1^+^ macrophages in wound healing processes.

## Results

### LYVE-1^+^ cells are a dominant population of tissue-resident macrophages in the dental pulp under steady-state conditions

**Figure 1:**
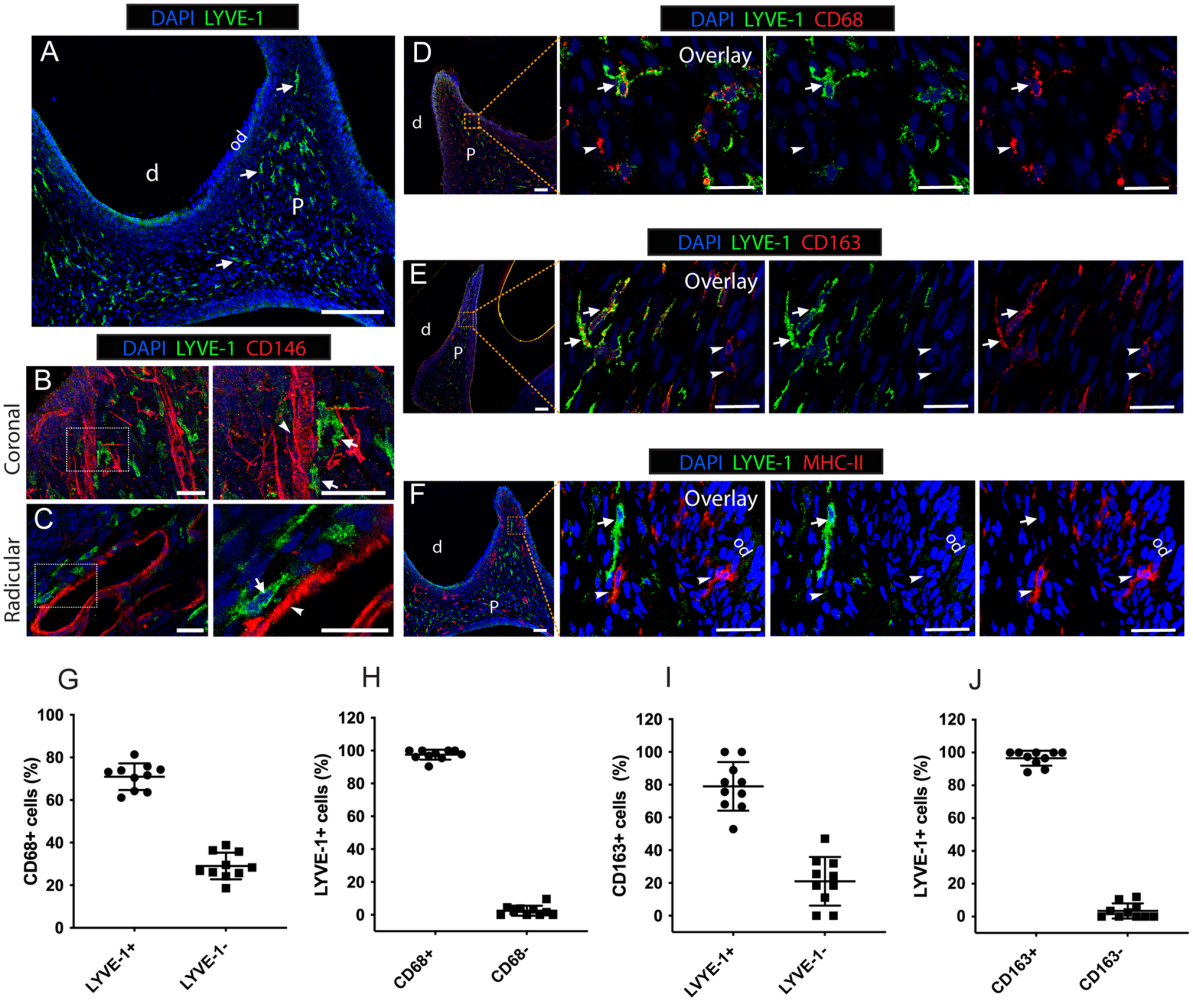
The phenotype of resident LYVE-1^+^ macrophages in dental pulp tissue of rats (8 weeks old). (**A**) Immunofluorescence staining of LYVE-1 in rat molar sections. Arrows: LYVE-1^+^ macrophages. (**B**,**C**). Double immunofluorescence staining of LYVE-1 (green) and endothelial cells marker CD146 (red) in rat molar sections. Arrows: LYVE-1^+^ macrophages; arrowheads: blood vessels. (**D**) Double immunofluorescence staining of LYVE-1 (green) and pan-macrophage marker CD68 (red) in rat molar sections. Arrows: LYVE-1^+^CD68^+^ macrophages; arrowheads: LYVE-1^-^CD68^+^ macrophages. (**E**) Double immunofluorescence staining of LYVE-1 (green) and M2 macrophage marker CD163 (red) in rat molar sections. Arrows: LYVE-1^+^CD163^+^; arrowheads: LYVE-1^−^CD163^+^ macrophages. (**F**) Double immunofluorescence stained of LYVE-1 (green) and antigen-presenting cell marker MHC-II (red) in rat molar sections. Arrows: LYVE-1^+^MHC-II^−^ macrophages; arrowheads: LYVE-1^-^MHC-II^+^ macrophages. (**G**–**J**). Percentages of macrophage subsets by cell counting (mean ± SD; n = 10). Images are representative of at least 3–4 different samples for each examined condition. *d* dentin, *od* odontoblast layer, *p* dental pulp. Scale bar 200 µm (**A**) and 50 µm (**B**–**F**).

Under a steady state, immunofluorescence-stained sections of 8-week-old rat molars showed numerous LYVE-1^+^ cells throughout the dental pulp tissue, to the exclusion of the odontoblastic layer (Fig. 1A). LYVE-1^+^ cells preferentially colonized close to blood vessels labeled with a vascular endothelial cell marker, CD146^24^, in both radicular and coronal pulp (Fig. 1B,C). To phenotypically characterize these LYVE-1^+^ cells, we performed double immunofluorescence staining for LYVE-1 and a pan-macrophage marker, CD68^25,26^, an M2 macrophage marker, CD163^26,27^, or an antigen-presenting cell marker, MHC-II^21^. Most LYVE-1^+^ cells were positive for CD68 and CD163 (Fig. 1D,E), while they were not labeled with MHC-II (Fig. 1F). Cell counting revealed that 97.5% ± 2.9% of LYVE-1^+^ cells were CD68-positive and accounted for 68.7% ± 11.1% of the CD68^+^ population (Fig. 1G,H). CD163 was expressed in 96.5% ± 4.4% of LYVE-1^+^ cells, while the proportion of LYVE-1^+^ cells among CD163^+^ cells was 79.0% ± 14.0% (Fig. 1I,J). Collectively, the LYVE-1^+^ population was a dominant subset of M2 macrophages resident in the dental pulp under steady-state conditions and was distinct from MHC-II^+^ antigen-presenting cells.

### LYVE-1^+^ tissue-resident macrophages are seeded in the dental papilla before birth

**Figure 2:**
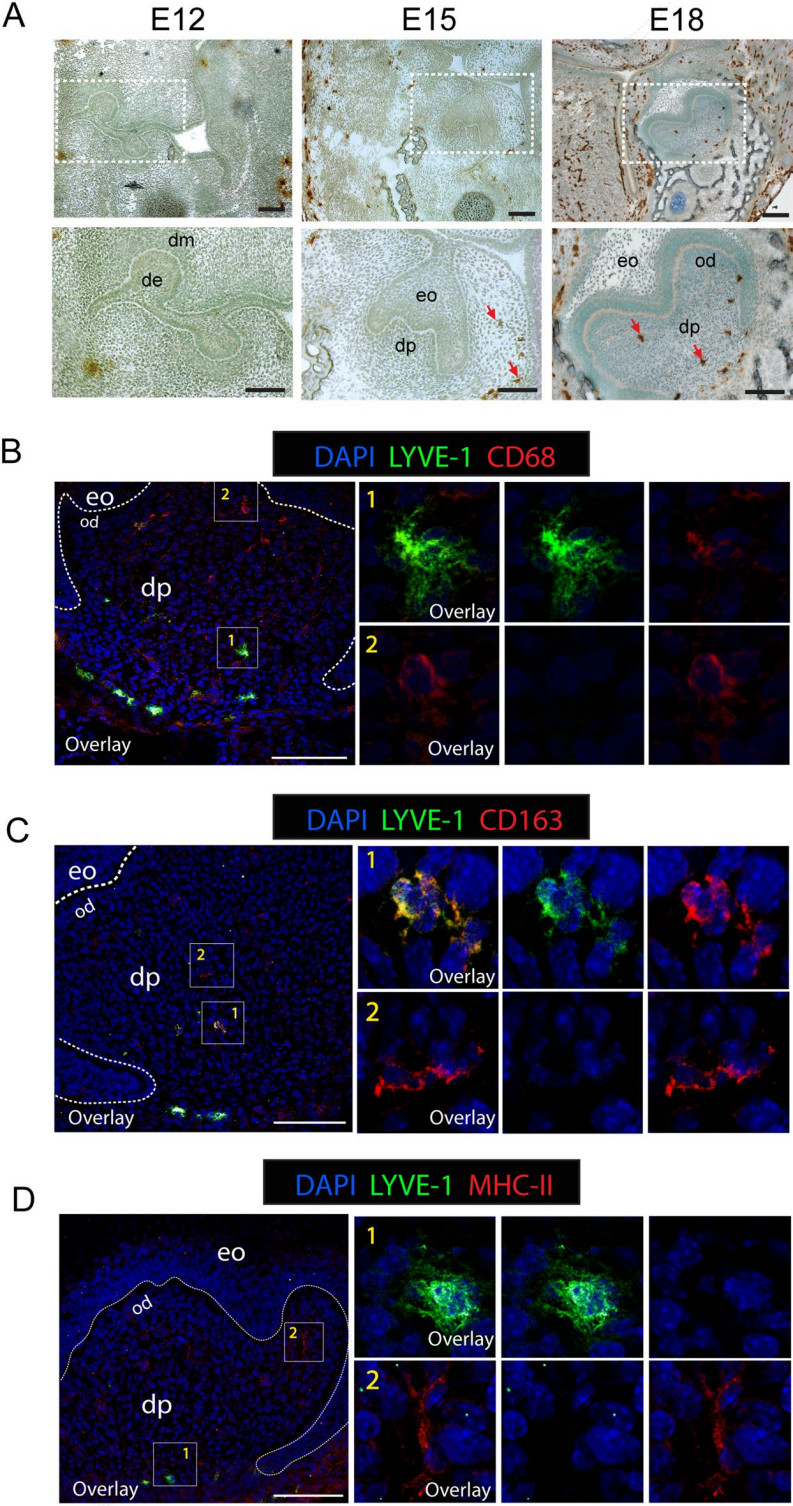
LYVE-1^+^ macrophages colonized in the dental papilla during embryonic tooth development. (**A**) Immunoperoxidase staining of LYVE-1 at embryonic days (E) 12, 15, and 18 in mouse embryo sections. Arrows: LYVE-1^+^ macrophages. (**B**) Double immunofluorescence staining of LYVE-1 (green) and pan-macrophage marker CD68 (red) at E18 in mouse embryo sections. (1) and (2) show high magnification of LYVE-1^+^CD68^+^ macrophages and LYVE-1^−^CD68^+^ macrophages, respectively. (**C**) Double immunofluorescence staining of LYVE-1 (green) and M2 macrophage marker CD163 (red) at E18 in mouse embryo sections. (1) and (2) show high magnification of LYVE-1^+^CD163^+^ macrophages and LYVE-1^−^CD68^+^ macrophages, respectively. (**D**) Double immunofluorescence staining of LYVE-1 (green) and antigen-presenting cell marker MHC-II (red) at E18 in mouse embryo sections. (1) and (2) show high magnification of LYVE-1^+^MHC^−^ macrophages and LYVE-1^−^CDMHC^+^ macrophages, respectively. Images are representative of at least 3–4 different samples for each condition examined. *De* dental epithelium, *dm* dental mesenchyme, *eo* enamel organ, *od* odontoblast layer, *dp* dental papilla. Scale bar 100 µm.

Most tissue-resident macrophage populations are established during embryonic development^7^. To evaluate whether the dental papilla is populated by LYVE-1^+^ macrophages before birth, we examined mouse first molars at embryonic days (E) 12, 15 and 18. Immunostaining for LYVE-1 showed that LYVE-1^+^ cells were absent from dental papilla from the bud stage (E12) to the cap stage (E15), and started to be observed in the middle of the dental papilla at the bell stage (E18) of tooth development (Fig. 2A). Double immunofluorescence staining for LYVE-1 and one of CD68, CD163, or MHC-II at E18 revealed that these LYVE-1^+^ cells were co-immunoreactive for both CD68 and CD163 (Fig. 2B,C) but negative for MHC-II (Fig. 2D). Additionally, at this stage, LYVE-1^−^/CD68^+^, LYVE-1^−^/CD163^+^, and LYVE-1^−^/MHC-II^+^ cells were scattered in the subodontoblastic layer (Fig. 2B–D). Collectively, these findings revealed that embryonic-derived LYVE-1^+^ macrophages were colonized in the dental papilla from the bell-stage of tooth development and exhibited an identical phenotype to LYVE-1^+^ macrophages found in the adult dental pulp.

### LYVE-1^+^ macrophages initially disappear and latterly recolonize in the dental pulp in response to dentinal cavity preparation

**Figure 3:**
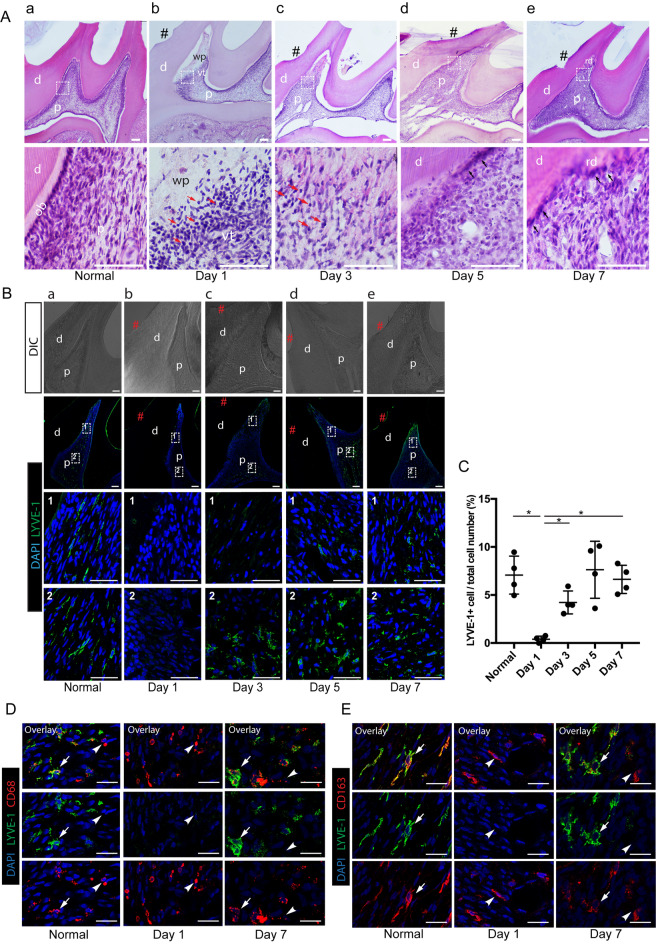
Distribution of LYVE-1^+^ cells in dental pulp after cavity preparation. (**Aa**–**e**) Rat molar sections at different time points after cavity preparation. Hematoxylin and eosin staining. Lower panels show a high magnification view of the boxed area in the corresponding upper panels. # indicates the cavity; red arrows, inflammatory cells; black arrows, odontoblast-like cells. (**Ba**–**e**) Immunofluorescence staining of LYVE-1 in rat molar sections at different time points after cavity preparation. Two regions selected for observation were: (1) the region in the mesial pulp horns beneath the cavity; and (2) the region in the mesial coronal portion distant from the cavity. # indicates the cavity. (**C**) Number of LYVE-1^+^ cells in the dental pulp after cavity preparation (mean ± SD). Four randomly selected representative images of the pulp tissue beneath the cavities were used for cell counting. Data were statistically analyzed by Brown-Forsythe/Welch one-way ANOVA. *p < 0.05. (**D**) Double immunofluorescence staining of LYVE-1 (green) and pan-macrophage marker CD68 (red) after cavity preparation in rat molar sections. White arrows: LYVE-1^+^CD68^+^ macrophages, white arrowheads: LYVE-1^−^CD68^+^ macrophages. (**E**) Double immunofluorescence staining of LYVE-1 (green) and M2-macrophage marker CD163 (red) after cavity preparation in rat molar sections. Arrows: LYVE-1^+^CD163^+^ macrophages, arrowheads: LYVE-1^−^CD163^+^ macrophages. Scale bar 150 µm (**A**), 100 µm (**B**), and 50 µm (**D**,**E**). *d* dentin, *p* dental pulp, *ob* odontoblast, *wp* wounded pulp, *vp* vital pulp, *rd* reparative dentin. Images are representative of at least 3–4 different samples for each condition examined.

Inflammation is one of the main factors to modulate the properties of tissue-resident macrophages by making them motile and changing the niche in which they reside^5^. To elucidate whether the inflammation status of the dental pulp impacts the distribution of the LYVE-1^+^ population, we performed cavity preparation without pulp exposure on rat molars, which can cause damage to the tissue beneath the cavity and subsequently trigger reversible inflammation. The inflammation status and wound-healing stages of the dental pulp after cavity preparation were histologically examined by hematoxylin and eosin (H&E) staining (Fig. 3Aa–e). On day 1 post-cavity preparation, the pulp tissue just beneath the cavity showed a necrotic area with sparse cellular components and an accumulation of infiltrated inflammatory cells along the border between the necrotic and vital pulp (Fig. 3Ab). From day 3 to day 7 after cavity preparation, inflammatory cells decreased their number in the wounded pulp tissue (Fig. 3Ac–e). Coinciding with the resolution of inflammation, dental pulp cells were repopulated in the wounded area on day 3 post-cavity preparation, suggesting the early stage of wound healing (Fig. 3Ac). From day 5 to day 7 post-cavity preparation, newly differentiated odontoblast-like cells lined the dentin wall and produced a thin layer of reparative dentin beneath the cavity (Fig. 3Ad,e) (Supplementary Fig. 2).

We next examined the distribution of LVYE-1^+^ macrophages in the injured pulp after cavity preparation by immunofluorescence staining. At steady state, LYVE-1^+^ macrophages were widely distributed throughout the dental pulp (Fig. 3Ba). On the first day after cavity preparation, a drastic loss of LYVE-1^+^ cells was observed in the affected pulp tissue (Fig. 3Bb). Notably, despite an almost complete loss in the affected area of the pulp at 1 day post-cavity preparation, LYVE-1^+^ cells could be detected in the pulp tissue distant from the cavity (Supplemental Fig. 1). At 3 days post-cavity preparation, even though several LYVE-1^+^ cells were detected in the central coronal pulp, they were still absent from the tissue beneath the cavity (Fig. 3Bc). On day 5 post-cavity preparation, many LYVE-1^+^ cells were observed in the central coronal pulp, and a small number of these cells were detected near the cavity (Fig. 3Bd). At 7 days post-cavity preparation, recovery of the LYVE-1^+^ cell population was evident by their extensive presence in the dental pulp, including in the tissue adjacent to the cavity (Fig. 3Be). Quantitative analysis of LYVE-1^+^ cells confirmed that the number of these cells was significantly decreased on day 1 and gradually increased to a level comparable to the control on day 7 post-cavity preparation (Fig. 3C). Double immunofluorescence staining for LYVE-1 and another macrophage marker revealed that, despite the disappearance of LYVE-1^+^/CD68^+^ and LYVE-1^+^/CD163^+^ macrophages, many LYVE-1^−^/CD68^+^ and LYVE-1^−^/CD163^+^ macrophages were still present in the tissue beneath the cavity on day 1 post-cavity preparation (Fig. 3D,E). Collectively, the distribution and density of the LYVE-1^+^ population in the dental pulp highly depends on the inflammation status: they disappeared at the acute inflammatory phase and recovered at the healing phase when the tissue remodeling progressed.

### Pro-/anti-inflammatory stimuli alter the expression of LYVE-1 in RAW264.7 cells

**Figure 4:**
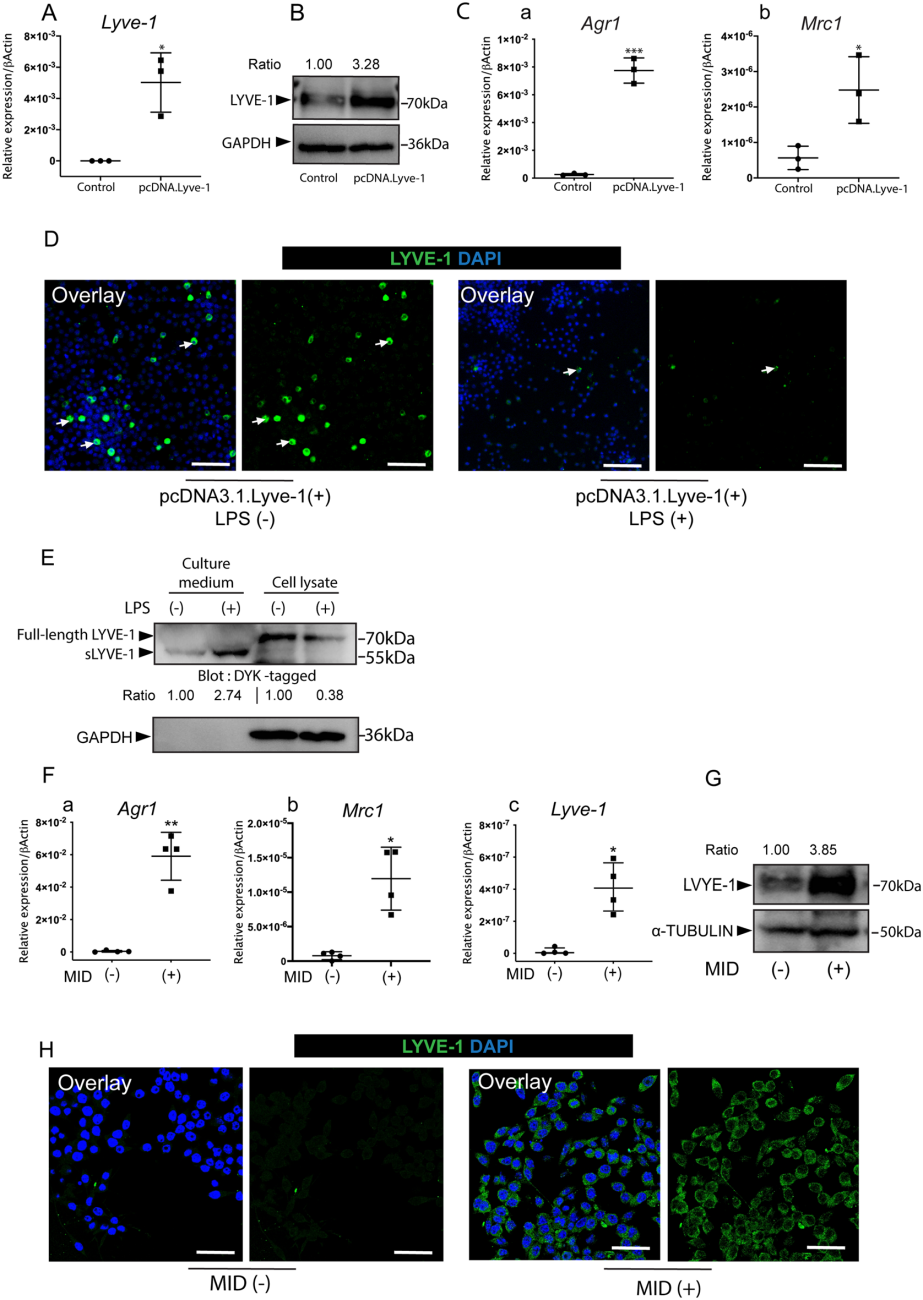
Pro-/anti-inflammatory stimuli orchestrate the expression of LYVE-1 in RAW264.7 cells. A *Lyve1-*expression vector (Lyve1_OMu20286D_pcDNA3.1+/C-(K)-DYK) was transfected into RAW264.7 cells to generate *Lyve1*-expressing macrophages. (**A**) RT-PCR analysis of *Lyve1* in RAW264.7 cells and *Lyve1*-expressing macrophages (mean ± SD, n = 3). (**B**) LYVE-1 protein expression of cell lysates collected from RAW264.7 cells and *Lyve1*-expressing macrophages as revealed by western blotting. (**C**) RT-PCR analysis of M2 macrophage markers (*Agr1, Mrc1)* in RAW264.7 cells and *Lyve1-*expressing macrophages (mean ± SD, n = 3). (**D**) *Lyve1-*expressing macrophages were treated with LPS (100 ng/ml) for 4 h. Immunofluorescence staining of LYVE-1 was used to detect LYVE-1-positive macrophages (arrows). (**E**) Cell lysates and culture media from LPS-treated and -untreated *Lyve1*-expressing macrophages were analyzed by western blotting with anti-DYK antibody. sLYVE-1, soluble LYVE-1. (**F**–**H**) M2 polarization of RAW264.7 cells induced by MID for 7 days. (**F**) RT-PCR analysis of M2 macrophage markers (*Agr1* and *Mrc1*) (mean ± SD, n = 4) and *Lyve1* (median with interquartile range, n = 4) in RAW 264.7 cells and M2-polarized cells. (**G**) Cell lysates collected from RAW 264.7 cells and M2 polarized cells were analyzed by western blotting with anti-LYVE-1 antibody. (**H**) Immunofluorescence staining of LYVE-1 in RAW 264.7 cells and M2 polarized cells. Data were collected from three independent experiments, and statistically analyzed by unpaired Welch's t-test (**A**,**Fa**,**b**), unpaired Student’s *t*-test (**Ca**,**b**), or Mann–Whitney U test (**Fc**). ∗*p* < 0.05, ∗∗*p* < 0.01, and ∗∗∗*p* < 0.0001. Scale bar 100 µm (**D**) and 50 µm (**H**).

In response to cavity preparation, the number of LYVE-1^+^ macrophages significantly decreased during the acute phase of inflammation. Therefore, we hypothesized that pro-inflammatory stimuli abolish the expression of LYVE-1. To test this hypothesis, the murine monocytic cell line RAW264.7 was transfected with a LYVE-1 expression vector (pcDNA.3.1-DYK.LYVE-1) to generate *Lyve1-*expressing macrophages followed by lipopolysaccharide (LPS; 100 ng/ml) stimulation for 4 h. Expression of *Lyve1* mRNA and protein was increased significantly in *Lyve1*-expressing macrophages (Fig. 4A,B). Additionally, *Lyve1-*expressing macrophages showed strong M2 polarization, as revealed by their high mRNA expression of typical M2 macrophages markers arginase 1 (*Agr1*) and mannose receptor C type 1 (*Mrc1*) (Fig. 4C). Immunofluorescence staining revealed that LYVE-1-positive cells disappeared after LPS stimulation (Fig. 4D). As a transmembrane glycoprotein, the ectodomain of LYVE-1 can undergo proteolytic cleavage that produces soluble LYVE-1 (sLYVE-1) in response to different stimuli^28,29^. We considered the possibility that LPS induced ectodomain shedding of LYVE-1, which led to the loss of cell surface LYVE-1 and the accumulation of sLYVE-1 in the culture medium. Western blotting of cell lysates revealed decreased LYVE-1 protein levels upon LPS stimulation, whereas western blotting of culture media collected from LPS-treated cells showed a detectable band of sLYVE-1, which was shorter (55 kDa) compared with full-length LYVE-1 (70 kDa) (Fig. 4E).

As we demonstrated, LYVE-1^+^ cells in the dental pulp expressed M2 macrophage markers and reappeared during the healing phase when the inflammation subsided. We next investigated the possibility that M2 polarization induces upregulation of LYVE-1. To induce M2 polarization, RAW264.7 cells were treated with a cocktail of macrophage-colony stimulating factor (M-CSF), interleukin-4 (IL-4), and synthetic glucocorticoid dexamethasone (MID) for 7 days. MID stimulation increased the mRNA levels of M2-markers *Arg1* and *Mrc1*, along with *Lyve1* (Fig. 4F). Western blotting and immunofluorescence staining further confirmed the increased protein levels of LYVE-1 in MID-treated RAW264.7 cells (Fig. 4G,H). Taken together, these results revealed that pro-inflammatory signals abolish the expression of LYVE-1, whereas anti-inflammatory signals activated RAW264.7 cells to the M2 direction, resulting in the upregulation of LYVE-1. We therefore suggest that the expression of LYVE-1 highly depends on the pro-/anti-inflammatory stimuli in the local environment where the macrophages reside.

### *Lyve1*-expressing macrophages promote the migration and tube formation of human umbilical vein endothelial cells

**Figure 5:**
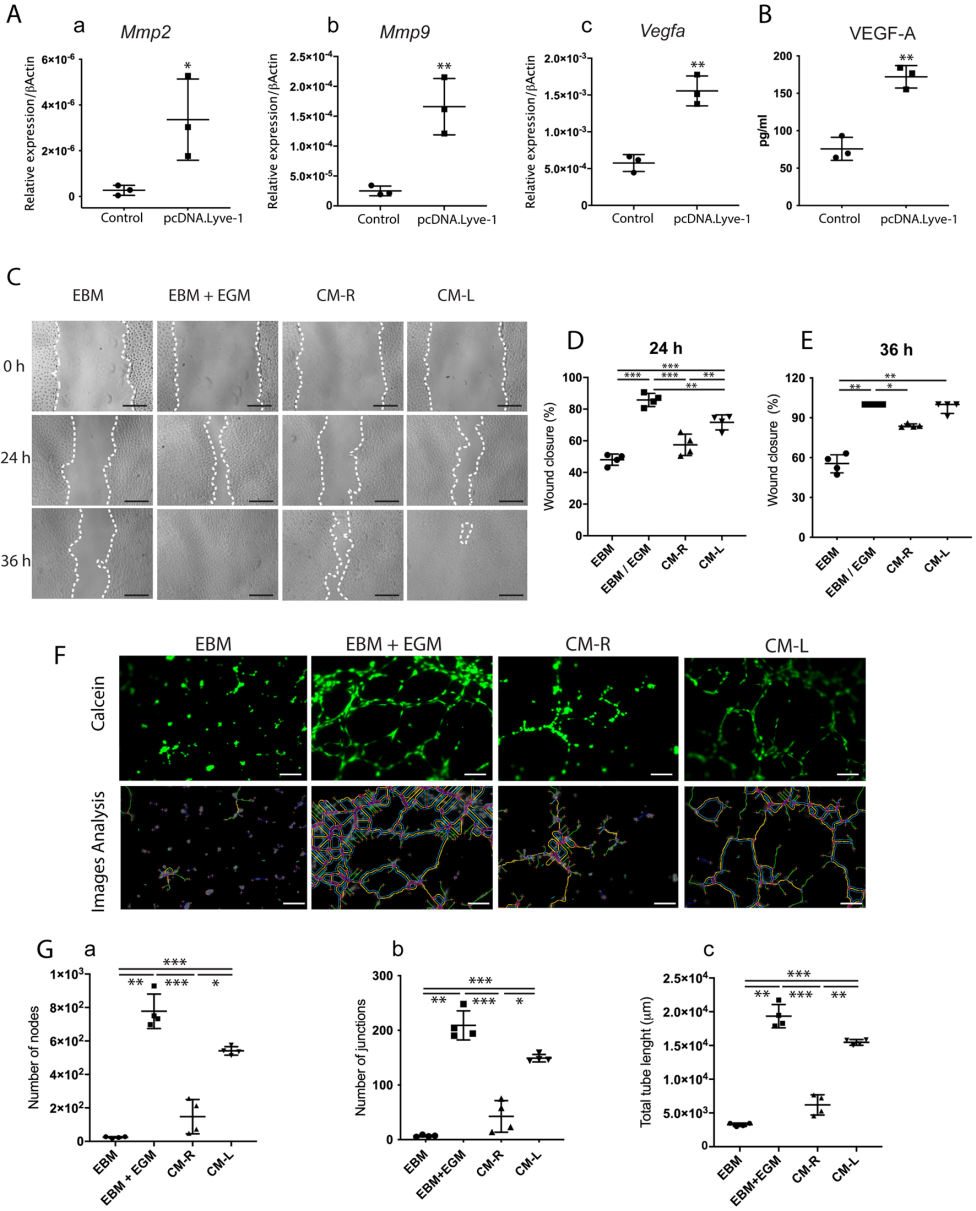
LYVE-1-expressing macrophages promote the migration and tube formation of human umbilical vein endothelial cells (HUVECs). A *Lyve1-*expression vector was transfected into RAW264.7 cells to generate *Lyve1*-expressing macrophages. (**A**) RT-PCR analysis of angiogenesis-related genes (*Mmp2, Mmp9*, *and Vegfa*) in RAW264.7 cells and *Lyve1*-expressing macrophages (mean ± SD, n = 3). (**B**) Quantification of VEGF-A in conditioned medium from *Lyve1*-expressing macrophages (CM-L) and RAW264.7 cells (CM-R) by ELISA (mean ± SD, n = 3). (**C**) Scratch wound healing test for HUVECs. The confluent cell monolayer of HUVECs was scratched with 200-μl pipette tips. Conditioned media (CM-L, CM-R), EBM-2 supplemented with EGM-2 (EBM/EGM), or serum-free EBM-2 (EBM) were added to culture plates. (**D**,**E**) Percentages of closure of scratched area at 24 h (mean ± SD, n = 4) and 36 h (median with interquartile range) after the initial scratch. Scratched areas were measured using an optimized plugin for ImageJ. (**F**) HUVEC tube formation assay. HUVECs were resuspended in conditioned media (CM-R, CM-L), EBM/EGM, or EBM. Cell suspension was seeded onto the solidified ECM gel and incubated for 6 h. Lower panels show the detection of tube-like structures of the corresponding upper panels using ImageJ. (**G**) The numbers of nodes and junctions and total tube length were quantified by ImageJ (mean ± SD, n = 4). Scale bars 300 µm (**C**) and 100 µm. Data were collected from 2–3 independent experiments to obtain consistent results. Data were statistically analyzed by unpaired Student’s *t*-test (**Aa–c**,**B**), one-way ANOVA and Tukey's post hoc test (**D**), Kruskal–Wallis test and Dunn's post hoc test (**E**), or Brown-Forsythe and Welch ANOVA test and Dunnett's T3 post hoc test (**Ga–c**). ∗*p* < 0.05, ∗ *p* < 0.01, and ∗∗∗*p* < 0.0001.

As a dominant population of tissue M2 macrophages that repopulates dental pulp in the healing phase after injury, LYVE-1^+^ macrophages potentially play an important role in wound healing. To examine this hypothesis, *Lyve1-*expressing macrophages were generated from RAW264.7 cells as described above. Overexpression of LYVE-1 resulted in a significant increase in matrix metalloproteinase 2 (*Mmp2*), MMP-9 (*Mmp9*), and vascular endothelial growth factor A (*Vegfa*) mRNA expression (Fig. 5A). In addition, higher VEGF-A protein production was detected in *Lyve1-*expressing macrophages compared with the control (Fig. 5B).

To further examine the role of *Lyve1-*expressing macrophages in wound healing, scratch wound healing and tube formation assays were conducted using conditioned media from *Lyve1-*expressing macrophages (CM-L) and RAW264.7 cells (CM-R). Endothelial cell growth basal medium-2 (EBM-2; Lonza, Walkersville, MD, Japan), with or without EGM-2 (endothelial cell growth medium-2; SingleQuot kit; Lonza, Walkersville, MD, USA) and endothelial cell growth factor component, were used as positive and negative controls, respectively. The results of the scratch wound healing assay showed that CM-L promoted the migration of human umbilical vein endothelial cells (HUVECs) to the scratched area faster than EBM and CM-R, whereas EBM supplemented with EGM (EBM/EGM) showed the greatest improvement in the HUVEC migration (Fig. 5C). At twenty-four hours after the initial scratch, HUVECs treated with CM-L closed approximately 71% of the original scratched areas compared with 57% and 48% in CM-L- and EBM-treated cells, respectively (Fig. 5D). The highest closure of the scratched area was observed in EBM-/EGM-treated cells used as the positive control (Fig. 5E). After 36 h, CM-L was comparable to EBM/EGM by promoting complete closure, while the scratched area still remained in the EBM (55%) or CM-R (84%) groups (Fig. 5E). The results of the tube formation assay revealed that treatment with CM-L promoted the physical organization of HUVECs into tube-like structures after 6 h (Fig. 5F). HUVECs treated with EBM/EGM showed the highest number of nodes and junctions and total vessel length compared with all experimental groups, whereas treatment with CM-L led to a significantly higher number of nodes and junctions and total vessel length compared with CM-R and EBM (Fig. 5G). Thus, *Lyve1*-expressing macrophages promoted the migration and tube formation of HUVECs. These findings suggest the potential role of LYVE-1^+^ macrophages in the wound healing process by providing appropriate angiogenic factors such as matrix metalloproteinases and growth factors.

## Discussion

In the present study, we identified a population of tissue-resident M2 macrophages expressing LYVE-1 in dental pulp. During embryogenesis, their initial colonization was detected in the dental papilla around the bell stage of tooth development. As the main tissue resident macrophage population of the dental pulp, they exhibited a certain degree of dynamism in response to pulpal inflammation induced by cavity preparation. In vitro data supported our hypothesis that the expression of *Lyve1* highly depends on pro-/anti-inflammation signals. We further found that *Lyve1*-expressing macrophages promoted the migration and tube formation of HUVECs. On the basis of these findings, we propose that LYVE-1^+^ macrophages in the dental pulp rapidly respond to microenvironment changes and play an important role in supporting wound healing processes.

Under homeostatic conditions, LYVE-1^+^ macrophages are found in many different tissues, including sclera, lung, skin, heart mesentery, synovial membrane, kidney, adipose tissue, thymus, lymph node, and aorta^13,14,30^ The phenotype of LYVE-1^+^ macrophages has been reported so far. LYVE-1^+^ cells expressing M2-associated markers CD163 and CD206 are a predominant population of tissue-resident macrophages in the artery^14^. Similarly, LYVE-1^+^ macrophages found in the mammary gland are CD206-positive^31^. A recent study also demonstrated the presence of LYVE-1^+^ macrophage populations that are located in close proximity to capillaries in the lung, skin, and heart, and that express a high level of CD206 but are negative for MHC-II^13^. Consistent with these findings, LYVE-1^+^ macrophages in the normal dental pulp preferentially colonized tissue adjacent to capillaries, expressed CD163, and were distinct from MHC-II^+^ antigen-presenting cells. The unique functions of LYVE-1^+^ macrophages in maintaining tissue homeostasis under steady-state conditions have been reported. For example, LYVE-1^+^ macrophages prevent arterial stiffness and collagen deposition^16^, restrain tissue fibrosis^13^, and modulate extracellular matrix turnover^31^. It is plausible that pulpal LYVE-1^+^ macrophages also play an important role in supporting dental pulp homeostasis because these cells were distributed throughout the dental pulp tissue under steady-state conditions and shared a similar phenotype to other LYVE-1^+^ macrophage populations found in different tissues.

Most tissue-resident macrophage populations are derived from embryonic progenitors that persist in the tissues and maintain themselves locally throughout adulthood^4,7,32,33^. The existence of embryonic-derived LYVE-1^+^ macrophages has been observed in the early stage of tissue development. For example, during mouse embryogenesis, CD68^+^/LYVE-1^+^ macrophages invade the fetal heart around E14.5 and give rise to resident macrophages of the adult heart^32,34^. In line with these findings, we showed here that LYVE-1^+^ macrophages started to colonize the dental papilla around E18 and were phonetically identical to LYVE-1^+^ macrophages found in the mature pulp tissue. Therefore, we propose the concept that embryonic-derived LYVE-1^+^ macrophages potentially persist in dental pulp prior to birth and contribute to the LYVE-1^+^ macrophage population in adulthood.

Tissue-resident macrophages are highly plastic immune cells due to their ability to adapt to extrinsic factors derived from the tissue microenvironment^35^. Inflammation stimuli often result in the partial depletion of tissue-resident macrophage populations, which has been described as the macrophage disappearance reaction^9,36^. When the inflammation subsides, tissue niches return to steady-state conditions along with the recovery of tissue-resident macrophage populations^9^. For example, the number of LYVE-1^+^-resident arterial macrophages decreases immediately after LPS exposure and subsequently rebounds to the level observed during steady-state conditions after 1 week^18^. In line with these findings, we observed the disappearance of LYVE-1^+^ cells in the dental pulp 1 day after cavity preparation and their recovery to normal levels within a week. The disappearance reaction of tissue-resident macrophages is possibly associated with their immigration to draining lymphatics for antigens processing, necroptotic cell death as the result of tissue damage, diminishment of LYVE-1 protein expression, and/or phenotypical and functional switching in response to changes in tissue niches^2,9,37,38^. However, functional enrichment analyses of gene expression profiling have revealed that genes upregulated in LYVE-1^+^ macrophages are not enriched for term-related antigen processing compared with LYVE-1^−^ macrophages in fat tissue^14^. Notably, cavity preparation in our model caused a necrotic wound that was limited to the peripheral area of the pulp tissue beneath the cavity and that exhibited a severe loss of LYVE-1^+^ macrophages. Hence, the disappearance of LYVE-1^+^ macrophages in the dental pulp after cavity preparation is attributed not to their migration to the lymph nodes but rather to cell death, diminishment of LYVE-1 protein expression, or their character switching in response to the signals in the local tissue microenvironment. Evidence supporting this notion includes the fact that LYVE-1 is a type I transmembrane glycoprotein that can undergo ectodomain shedding to form sLYVE-1 in response to certain signals including inflammation^28,29^. Shedding of LYVE-1 is observed in inflamed lymphatic vessels^28^. Moreover, our in vitro findings revealed the loss of cell surface LYVE-1 expression and the accumulation of sLYVE-1 in the culture medium after 4 h of LPS stimulation. Therefore, we propose the possibility that LYVE-1^+^ macrophages in inflamed pulp induced by cavity preparation can undergo shedding of its ectodomain and become LYVE-1^−^ macrophages.

The mechanism behind the renewal of LYVE-1^+^ macrophages in the dental pulp after cavity preparation remains unclear. It may be due to self-renewal by local proliferation of surviving macrophage subsets or the replenishment of recruited monocytes^39^. Indeed, we detected the remaining population of LYVE-1^+^ macrophages in the dental pulp after cavity preparation, distant from the cavities, suggesting that these cells can migrate toward the injured pulp tissue and give rise to the recovered population by local proliferation. Moreover, on recovery from the inflammatory phases, monocytes recruited to the tissue could identify homeostatic conditions for re-programming to an M2-like phenotype by Th2 cytokines (IL-4, IL-13), thereby becoming recovered tissue macrophages over time^40–42^. Our data also indicated that M2 macrophages activated by IL-4 upregulated LYVE-1 expression. We suggest that when inflammation is resolved, recruited monocytes can undergo reprogramming in response to anti-inflammatory cytokines in the local environment and replace the loss of original LYVE-1^+^ macrophages.

Macrophages are critical in all phases of the wound healing process, which occurs in three overlapping stages: (1) inflammation; (2) resolution of inflammation; and (3) tissue vascularization and regeneration^35,43^. Most tissue-resident macrophages show strong M2 polarization^44^, which are critical players in the final stages of wound healing^43,45^. While inflammatory M1 macrophages initially infiltrate the wound in an attempt to phagocytose and kill the invaders, anti-inflammatory M2 macrophages subsequently provide growth factors to support the healing process after the dangerous pathogens have been eliminated^11,46,47^. It has been reported that cavity preparation in rat molars resulted in the destruction of capillary networks in the subodontoblastic layer immediately after the operation, and subsequently, the density and thickness of blood vessels began to increase in the wounded pulp and reached a peak on postoperative day 3 to day 5^48^. Our data revealed that LYVE-1^+^ macrophages expressing M2 macrophage markers repopulate dental pulp after 3 days post-injury, which coincides with the vascularization and regeneration phases of the wound healing process. Therefore, it is plausible that LYVE-1^+^ macrophages may participate in promoting wound healing. Previously, LYVE-1^+^ macrophages have been reported to participate in the angiogenesis of adipose tissue by secreting MMP-7, MMP-9, and MMP-12^17^. We found that *Lyve1*-expressing macrophages generated from RAW264.7 cells showed upregulation of angiogenesis-related genes and produced a high level of VEGF-A. Furthermore, conditioned media from *Lyve1*-expressing macrophages promoted the migration and tube formation of HUVECs. Collectively, these findings suggest that LYVE-1^+^ macrophages orchestrate tissue repair during the healing phases after injury of dental pulp by promoting angiogenesis via the secretion of pro-angiogenic factors such as MMPs and VEGF-A.

A limitation of the present study is that we evaluated the biological function using *Lyve1*-expressing RAW264.7 cells, and not LYVE-1^+^ macrophages sorted from dental pulp tissue. Strictly speaking, these cells have different properties. However, Lyve1-expressing RAW264.7 cells also expressed M2 markers such as *Arg1* and *Mrc1*, suggesting that they share some similarities with LYVE-1^+^ macrophages existing in the dental pulp. Our findings contribute to the current understanding of LYVE-1^+^ macrophages, which are the predominant tissue-resident macrophage population in the dental pulp. However, their precise roles in the dental pulp during physiological and pathological conditions require further investigation. Recently, attention has been focused on pulp regeneration therapy in the field of endodontic treatment. In particular, induction of angiogenesis in newly formed tissue is critical^49^. Because we demonstrated the potentially pro-angiogenic ability of LYVE-1^+^ macrophages, targeting LYVE-1^+^ macrophages could be a promising therapeutic strategy for dental pulp regeneration.

## Conclusion

We revealed that LYVE-1^+^ macrophages in dental pulp mainly comprised M2-like tissue resident macrophages. After pulp injury induced by cavity preparation, they drastically disappeared at the initial inflammatory phase and repopulated the area at the resolution phase. In vitro, *Lyve1*-expressing macrophages generated from RAW264.7 cells increased the mRNA expression of angiogenic MMPs and secreted VEGF-A and promoted the migration and tube formation of HUVEC cells. These findings suggest that Lyve1^+^ macrophages play an important role in angiogenesis during wound healing and tissue remodeling of injured dental pulp tissue.

## Materials and methods

### Cell culture

The murine macrophage cell line, RAW264.7 (Riken Bioresource Research Center, Tsukuba, Japan), was incubated in Dulbecco’s modified Eagle medium (DMEM; Wako Pure Chemical Industries, Osaka, Japan) supplemented with 10% heat-inactivated fetal bovine serum (FBS; Thermo Fisher Scientific, Waltham, MA, USA) and 10% penicillin/streptomycin (Wako Pure Chemical Industries). Human umbilical vein endothelial cells (HUVECs; Promo Cell, Heidelberg, Germany) were cultured in Endothelial Basal Medium-2 (EBM2; Lonza) supplemented with Endothelial Cell Growth Medium-2 SingleQuots Kit (EGM-2; Lonza). All cells were cultured in standard conditions (37 °C, 5% CO_2_) and the culture medium was replaced every 3 days.

### Animal experiments and tissue preparation

All experiments were approved by the Animal Care and Use Committee of Tokyo Medical & Dental University (A2017-155A) and were conducted in accordance with relevant ethical guidelines and regulations. All animal experiments are reported in compliance with the ARRIVE guidelines^50^. Male Sprague Dawley rats (8 weeks old, n = 30) and pregnant C57BL/6J mice (n = 4) were obtained from CLEA Japan (Tokyo, Japan) and housed in standard conditions (22 °C, 55% relative humidity, artificial illumination). To generate the pulpal injury model, rats were anesthetized with an intraperitoneal injection of ketamine hydrochloride (50 mg/kg, Ketalar; Sankyo, Tokyo, Japan) and xylazine hydrochloride (20 mg/kg, Selactar; Bayer Yakuhin, Osaka, Japan). Cavities without pulp exposure were prepared on the mesial surface of the upper first molars of both sides with #1/2 round burs using a dental handpiece motor under a stereoscopic microscope (Dental Microscope Z; Mani, Tochigi, Japan). The thickness of remaining dentin was approximately 200 µm. On days 1, 3, 5, and 7 after cavity preparation, the rats were killed under carbon dioxide euthanasia. The maxilla was collected and fixed with 4% paraformaldehyde overnight. The samples were demineralized with 17% EDTA for 3 weeks and embedded in an embedding medium (Tissue-Tek OCT compound; Sakura Finetek, Torrance, CA, USA) for frozen sections. To obtain embryos, the pregnant mice were killed under carbon dioxide euthanasia. The heads of fetal mice were harvested at the desired developmental stages (E13, E15, and E18) and immediately embedded in OCT compound to make fresh frozen samples. Cryostat sections (10 µm) from rat maxilla and mouse embryos were prepared for histological evaluation.

### Immunohistochemistry and immunocytochemistry

Staining was performed as described previously^51^. Briefly, for immunofluorescence staining of frozen samples, sections were incubated with primary antibodies overnight at 4 °C. The antibodies used were anti-LYVE-1 (11-036, rabbit monoclonal; AngioBio, San Diego, CA, USA), anti-CD68 (MCA341GA, mouse monoclonal; Bio-Rad, Kidlington, UK), anti-CD163 (MCA342GA, mouse monoclonal; Bio-Rad), anti-MHC-II (MCA46R, mouse monoclonal; Bio-Rad), and anti-CD146 (MAB3250, mouse monoclonal; R&D Systems, MN, USA). This was followed by 1-h incubation with Alexa Fluor 488-conjugated anti-mouse IgG (ab150065; Abcam, Cambridge, UK) and/or Alexa Fluor 568-conjugated anti-rabbit IgG (ab175700; Abcam). Finally, nuclei were visualized by coverslipping with mounting media containing 4′,6′-diamidino-2-phenylindole nuclear stain (DAPI, Aqueous; Flouroshield, Abcam).

For cultured cells, 35-mm imaging dishes with a polymer coverslip bottom (Asahi Techno Glass, Shizuoka, Japan) were used for cell seeding. The cells were fixed with 4% paraformaldehyde and immunofluorescence staining was performed as described above. Histological analyses were performed using a confocal laser scanning microscope (Leica TCS-SP8, Leica Microsystems; Wetzlar, Germany) and LAS AF confocal software (Version 1.8.3, Leica Microsystems).

For immunoperoxidase staining, sections were fixed with 4% paraformaldehyde for 10 min at 4 °C. Endogenous peroxidase activity was blocked by incubating the sections in 0.3% H_2_O_2_ solution in PBS at room temperature for 10 min. The sections were incubated with an anti-LYVE-1 antibody (11-036; AngioBio) overnight at 4 °C, followed by 30-min incubation with biotinylated anti-rabbit IgG antibody (BA-1000; Vector Laboratories, Burlingame, CA, USA) and the avidin-biotin-peroxidase complex (R.T.U. Vectastain Universal ABC Kit, PK-7200; Vector Laboratories). The color reaction was performed using DAB substrate solution (ImmPACT DAB; Vector Laboratories). The sections were counterstained using methyl green (MUTO, Tokyo, Japan) and mounted using mounting medium (VectaMount; Vector Laboratories). Histology was observed under light microscopy (Axio Vert; Zeiss).

### Plasmid transfection

RAW264.7 cells were transfected with a *Lyve1* overexpression vector (Lyve1_pcDNA3.1+/C-(K)DYK; Clone ID OMu20286, GenScript, Piscataway, NJ, USA) using FuGENE HD (Promega, Madison, WI, USA) to generate *Lyve1*-expressing macrophages. All steps were performed in accordance to the manufacturer’s instructions. Briefly, RAW264.7 cells (2 × 10^5^ cells/ml) were seeded in 24-well plates 1day before transfection. FuGENE/pcDNA3.1.LYVE-1 complex mixture at a ratio of 3:1 was freshly prepared containing 0.5 μg expression vector. The mixture was added to the wells and the plates were incubated under standard conditions (37 °C, 5% CO_2_) for 24 h. Cells transfected with an enhanced green fluorescent protein (EGFP) expression vector (pMAX-EGFP; Lonza) were used as a control.

### LPS stimulation and M2 macrophage polarization

For LPS stimulation, *Lyve1*-expressing macrophages were cultured with or without LPS (100 ng/ml; *Escherichia coli* O111B4; Merck, Kenilworth, NJ, USA) for 4 h. For M2 macrophage polarization, RAW264.7 cells were seeded at a low density of 10^4^ cells/ml in 6-well plates followed by 7 days’ incubation in DMEM supplemented with 10% heat-inactivated FBS and MID, a mixture of M-CSF (100 ng/m; BioLegend, San Diego, CA, USA), IL-4 (10 ng/ml; PeproTech, East Windsor, NJ, USA), and dexamethasone (1000 U/ml; Wako Pure Chemical Industries). Culture medium was exchanged every 2 days.

### Preparation of conditioned media

RAW246.7 cells (10^5^ cells/ml) were seeded on 60-mm culture plates and transfected with *Lyve1* overexpression vector as described above. One day after transfection, the cells were washed thoroughly using PBS and replenished with serum-free EBM-2 for 24 h under standard conditions (37 °C, 5% CO_2_). Conditioned media from *Lyve1*-expressing macrophages (CM-L) or RAW246.7 cells (CM-R) were collected, centrifuged at 3000 rpm for 5 min at 4 °C to remove cell debris and stored at − 30 °C.

### Reverse transcription-quantitative polymerase chain reaction

Total RNA was extracted using a QuickGene RNA cultured cell kit S (Wako Pure Chemical Industries), and cDNA (300 ng) was synthesized using PrimeScript™ RT Master Mix (Takara Bio, Kusatsu, Japan). For reverse transcription-quantitative polymerase chain reaction (RT-qPCR), template cDNA was amplified with GoTaq qPCR Master Mix (Promega) using a CFX96 Real-Time qPCR System (Bio-Rad). For normalization of the template amount, gene expression was calculated in relation to *Actb* as a housekeeping gene. Specific primers are listed in Table 1.

**Table 1:** Primer sequences.

Genes	Forward (5′–3′)	Reverse (5′–3′)	Accession no
*Actb*	AGCTGTGCTATGTTGCTCTAGACTT	CACTTCATGATGGAATTGAATGTAG	NM_007393
*Lyve1*	TGTTGCTACGTGAAAAGGTATGTC	ATCAGCCTTCTCTTCCTTTACAACC	NM_053247.4
*Mrc1*	AGCAGCTATTCCCTTATGAAATTGAAG	AAAATAGTAGCAATGGCCATAGAAAGG	NM_008625.2
*Arg1*	CAGAGAAGGTCTCTACATCACAGAA	GTGTTCACAGTACTCTTCACCTCCT	NM_007483.3
*Mmp2*	CTTCCTGTTCAACGGTCGGGAATAC	CATGGTAAACAAGGCTTCATGGGG	NM_008610.3
*Mmp9*	TGGTCTTCCCCAAAGACCTGAAAAC	GTAGAGACTGCTTCTCTCCCATCAT	NM_013599.5
*Vegfa*	GAGAGCAACATCACCATGCAGATCA	GGCTCACAGTGATTTTCTGGCTTT	NM_009505.4

Accession numbers are available at https://www.ncbi.nlm.nih.gov/nucleotide/.

### Western blotting

Cells were lysed with a radioimmunoprecipitation assay buffer containing a protease inhibitor cocktail (cOmplete; Roche, Mannheim, Germany) and phosphatase inhibitor cocktail (PhosSTOP; Roche). Proteins were separated using 10% polyacrylamide ready-made gel (e-PAGEL, ATTO, Tokyo, Japan). After transfer to PVDF transfer membrane (Immobilon-P, Merck Millipore, Burlington, MA, USA) using a semi-dry transfer system (0.15 mA, 1 h; WSE-4040, ATTO), the blot was incubated with the following primary antibodies: anti-LYVE-1 (11-034, rabbit; AngioBio), anti-DYK-tagged (M185-7, mouse monoclonal; MBL, Nagoya, Japan), anti-α tubulin (PM054-7, rabbit polyclonal; MBL), and anti-GAPDH (M171-7, mouse monoclonal; MBL). Horseradish peroxidase-conjugated anti-rabbit IgG (W4011; Promega) and anti-mouse IgG (W4021; Promega) were used as secondary antibodies. For signal detection, Immobilon Western Chemiluminescent HRP Substrate (Merck Millipore) and a digital autoradiograph imaging system (LAS-3000; Fujifilm, Tokyo, Japan) were used. The relative density of bands was quantified using ImageJ (version 1.8.0; National Institute of Health, New York, NY, USA).

### Enzyme-linked immunosorbent assay

The protein level of VEGF-A in conditioned media was determined using DuoSet ELISA mouse VEGF (DY493; R&D Systems) in accordance with the manufacturer's instructions.

### Wound healing assay

HUVECs (3.0 × 10^5^ cells/ml) were seeded in 24-well plates in endothelial cell growth basal medium-2 (EBM-2; Lonza) containing EGM-2 (SingleQuot kit; Lonza). When HUVECs had formed a confluent cell monolayer, cells were starved in serum-free EMB-2 for 24 h. Cell monolayers were scratched with 200-μl pipette tips and carefully rinsed with PBS to create uncovered areas in the center of the cultured wells. Conditioned media (CM-L, CM-R), EBM-2 supplemented with EGM-2 (positive control), and serum-free EBM-2 (negative control) were added to culture plates. Images were captured immediately following media replacement and at 12 h and 36 h with a microscope (Axio Vert; Zeiss). The images were analyzed, and wound areas were measured using an optimized plugin for ImageJ to automatically recognize the wound healing size. The percentages of wound closure were calculated using the following equation:

$${\text{Wound closure }}\left( \% \right) = {1}00\left( {{\text{A}}_{{{\text{T}} = 0}} {-}{\text{A}}_{{{\text{T}} = \Delta {\text{t}}}} } \right)/{\text{A}}_{{{\text{T}} = 0}}$$

*A*_*T*=0_ is the initial wound area (μm^2^) and *A*_*T*=*Δt*_ is the wound area after 24 h or 36 h of the initial scratch (μm^2^).

### Tube formation assay

Fifty microliters ECM gel solution (Cell Biolabs, San Diego, CA, USA) was coated on 96-well plates followed by 30-min incubation at 37 °C for solidification. HUVECs were harvested and resuspended in conditioned media (CM-R, CM-L), EBM-2 supplemented with EGM-2 (positive control), and serum-free EBM-2 (negative control) at 10^5^ cells/ml. One hundred fifty microliters cell suspension was seeded onto the solidified ECM gel and incubated for 6 h. Endothelial tubes were labeled with Calcein AM (Cell Biolabs) and examined under a fluorescence microscope (Axio Vert; Zeiss). Several randomized images per well were captured. The numbers of nodes and junctions and total tube length were quantified by ImageJ (version 1.8.0; National Institute of Health).

### Cell quantification

Quantification of cell number was performed by manual cell counting of histological sections. The images used for cell counting were randomly captured at the mesial pulp horn area and mesial coronal pulp portion with a 40× objective lens and an acquisition resolution of 1024 × 1024 pixels. For each analysis, representative images (n ≥ 4) of each experimental group were used.

### Statistical analysis

Statistical analysis was performed using JASP (version 0.16, University of Amsterdam, Amsterdam, The Netherlands) and GraphPad Prism 7 (version 7.01; GraphPad Software Inc, California, USA). The normality was checked by Shapiro–Wilk’s test and the equality of variances was checked by Levene’s test. Data with normal distribution and equal variance were statistically analyzed by unpaired Student’s *t*-test (between 2 groups) or one-way ANOVA and Tukey's post hoc test (among 3 groups or more). Data with normal distribution and unequal variance were statistically analyzed by unpaired Welch's t-test (between 2 groups) or Brown-Forsythe/Welch one-way ANOVA test and Dunnett's T3 post hoc test (among 3 groups or more). Data with non-normal distribution were statistically analyzed by Mann–Whitney U test (between 2 groups) or Kruskal–Wallis test and Dunn's post hoc test (among 3 groups or more). *P* values of < 0.05 were considered statistically significant.

## Supplementary Information

Supplementary Legends.

Supplementary Figure 1.

Supplementary Figure 2.

Supplementary Figure 3.
