# Supplementary material for: Kinetics of LYVE-1-positive M2-like macrophages in developing and repairing dental pulp in vivo and their pro-angiogenic activity in vitro
Source: Sci Rep. 2022 Mar 25;12:5176. doi: 10.1038/s41598-022-08987-3 (PMC8956626; doi:10.1038/s41598-022-08987-3)
Supplement: Supplementary file 1 — Supplementary Legends. [file 41598_2022_8987_MOESM1_ESM.docx]

**Supplementary Figure legends:**

(Figure S1). Immunofluorescence staining of LYVE-1 on day 1 after cavity preparation in rat sections. (1): LYVE-1^+^ cells (green) had disappeared in the dental pulp beneath the cavity. (2): LYVE-1^+^ cells (green) were remaining in the central coronal portion of dental pulp. Cell nuclei were stained with DAPI (blue). # indicates the cavity, d, dentin; p, dental pulp. Scale bar = 100 µm.

(Figure S2). Immunoperoxidase staining of nestin in rat sections at day 5 and day 7 after dental cavity preparation. Lower panels show a high magnification view of the boxed area in the corresponding upper panels. # indicates the cavity; arrows, nestin positive cells; d, dentin; p, dental pulp; od, odontoblast layer. Scale bar = 120 µm.

(Figure S3). The images of full-length blots. (A) Uncropped images for immunoblots of Figure 4B, LYVE-1 protein expression of control-RAW264.7 cells and *Lyve1*-expressing macrophages. (B) Uncropped images for immunoblots of Figure 4E, LYVE-1 protein expression in cell lysates and culture media from LPS-treated and -untreated *Lyve1*-expressing macrophages. (C) Uncropped images for immunoblots of Figure 4G, LYVE-1 protein expression of control-RAW264.7 cells and MID-treated RAW264.7 cells. dashed red boxes, the cropped regions used in the main figures.
