# Supplementary figures and images for: Kinetics of LYVE-1-positive M2-like macrophages in developing and repairing dental pulp in vivo and their pro-angiogenic activity in vitro
Source: Sci Rep. 2022 Mar 25;12:5176. doi: 10.1038/s41598-022-08987-3 (PMC8956626; doi:10.1038/s41598-022-08987-3)

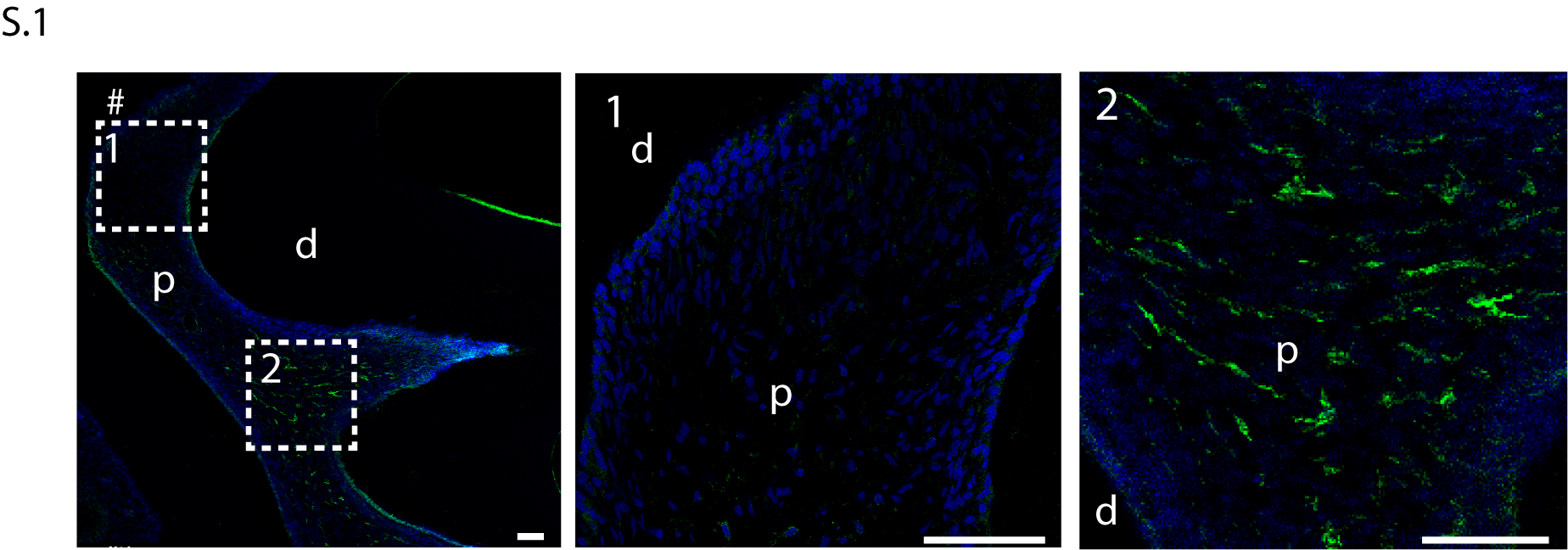

Supplement: Supplementary file 2 — Supplementary Figure 1. [file 41598_2022_8987_MOESM2_ESM.tif]

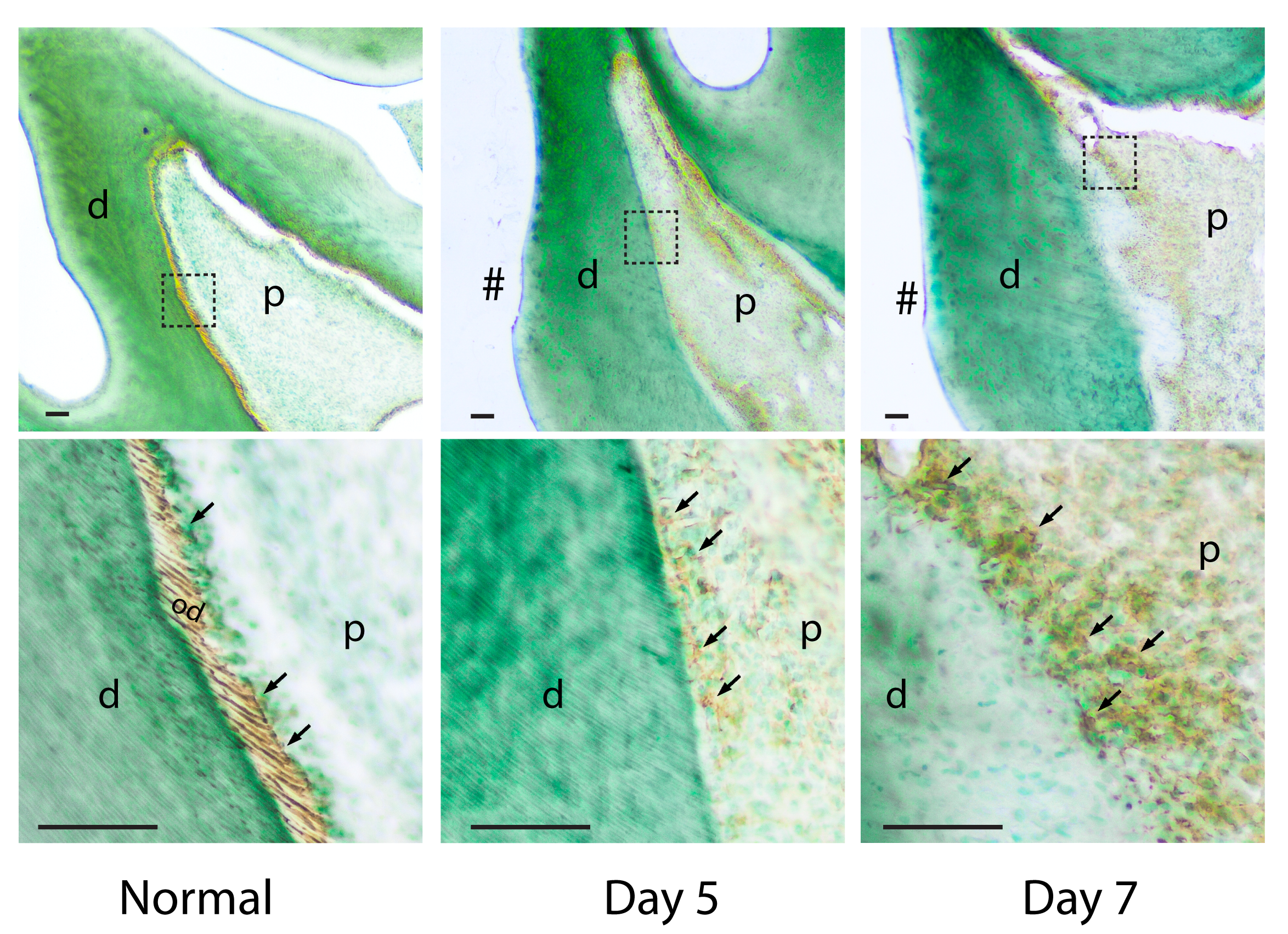

Supplement: Supplementary file 3 — Supplementary Figure 2. [file 41598_2022_8987_MOESM3_ESM.tif]

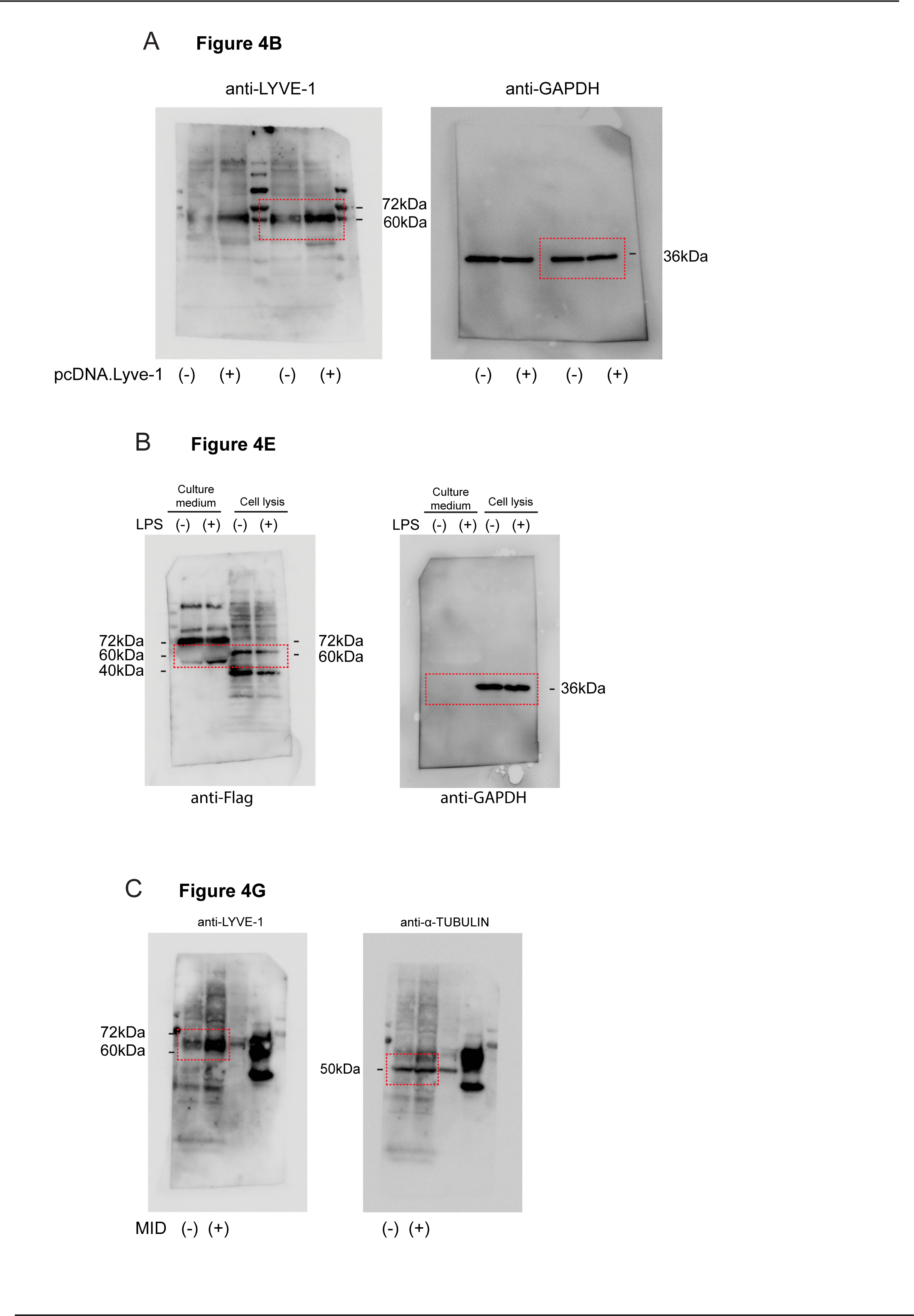

Supplement: Supplementary file 4 — Supplementary Figure 3. [file 41598_2022_8987_MOESM4_ESM.tif]
